# Associations between blood glucose-lipid levels and post-intravenous thrombolysis outcomes in stroke patients: a retrospective study utilizing logistic regression analysis

**DOI:** 10.3389/fneur.2026.1701394

**Published:** 2026-02-18

**Authors:** Liu He, Rong Li, Yan Liu, Xin Tan, Lei Wang, Xi Zhu, Qiang Zhou, Zhiyong Yang, Hua Liu

**Affiliations:** Department of Neurology, Affiliated Hospital of Southwest Jiaotong University, The Third People’s Hospital of Chengdu, Chengdu, Sichuan, China

**Keywords:** acute ischemic stroke, blood glucose-lipid levels, intravenous thrombolysis, logistic regression model, modified Rankin scale

## Abstract

**Objective:**

This study investigates the relationship between blood glucose and lipid profiles and the functional outcomes of acute ischemic stroke (AIS) patients treated with intravenous thrombolysis.

**Methods:**

We retrospectively analyzed data from AIS patients admitted to a tertiary hospital in Chengdu between September 2023 and March 2025. Patients were grouped according to post-thrombolysis modified Rankin scale (mRS) scores into good outcome (0–2) and poor outcome (3–6) categories. Multivariable logistic regression and nomogram modeling were used to identify predictors of 30- and 90-day outcomes.

**Results:**

The final predictive model included 11 variables, and the results showed higher age, smoking, diabetes, higher systolic blood pressure (SBP), higher diastolic blood pressure (DBP), higher total cholesterol (TC), lower high-density lipoprotein cholesterol (HDL), higher low-density lipoprotein cholesterol (LDL), higher haemoglobin A1c (HbA1c), and higher fasting plasma glucose (FPG) were independent risk factors. This model demonstrated robust predictive performance and accuracy across all timepoints (training dataset at 30 days, the AUC of 0.821, 95% CI: 0.765–0.877; training dataset at 90 days, the AUC of 0.871, 95% CI: 0.824–0.919).

**Conclusion:**

Blood glucose and lipid levels are linked to outcomes after intravenous thrombolysis in stroke. Despite model limitations, these modifiable parameters may support risk-stratified management, potentially improving functional outcomes post-thrombolysis.

## 1 Introduction

Ischemic stroke (IS), one of the leading global causes of disability and mortality, has consistently been a major research focus in neuroscience and public health concerning its epidemiological characteristics and risk factor management (1–3). Epidemiological data indicate that IS incidence is associated with demographic factors such as age, sex, ethnicity, and socioeconomic status, with men affected more often than women and over 60% of cases occurring in Asian populations (2, 4).

Dyslipidemia is a critical contributor to IS risk, primarily through the promotion of atherosclerosis (5–7). Conversely, HDL exerts protective effects by facilitating reverse cholesterol transport and reducing inflammation, with each 1 mmol/L increase associated with a 15–20% reduction in risk (6, 7). Furthermore, elevated triglycerides (TG) are associated with an increase in remnant lipoprotein particles and may contribute to a higher risk of IS through prothrombotic effects (8).

Despite the well-established role of statins in reducing LDL levels and preventing recurrent IS, lipid management faces significant challenges globally (9–11). Intravenous alteplase (rt-PA) is the standard therapy for AIS within 4.5 h of symptom onset, working by activating plasminogen and dissolving fibrin clots (12, 13). Clinical trials have shown that thrombolysis significantly improves the chance of achieving mRS ≤2 at 90 days compared to non-thrombolyzed patients (OR = 2.5–3.5) (14, 15). However, the rate of vascular re-occlusion post-thrombolysis is notably high, ranging from 14 to 34%, primarily associated with persistent *in-situ* thrombosis and local hypercoagulability (16). The efficacy and safety of thrombolytic therapy are highly dependent on the treatment time window (within 4.5 h of onset) and individual patient characteristics, including lipid levels, thrombus burden, comorbidities and the type and dosage of the thrombolytic agent used (17, 18). In recent years, clinical studies have focused on optimizing the efficacy and predicting outcomes of intravenous alteplase thrombolysis. However, the impact of glycemic and lipid abnormalities on post-thrombolysis neurological recovery remains controversial. This study aimed to evaluate whether admission glucose and lipid profiles independently predict functional outcomes following intravenous thrombolysis in AIS patients.

## 2 Methods

### 2.1 Study population

**Figure 1:**
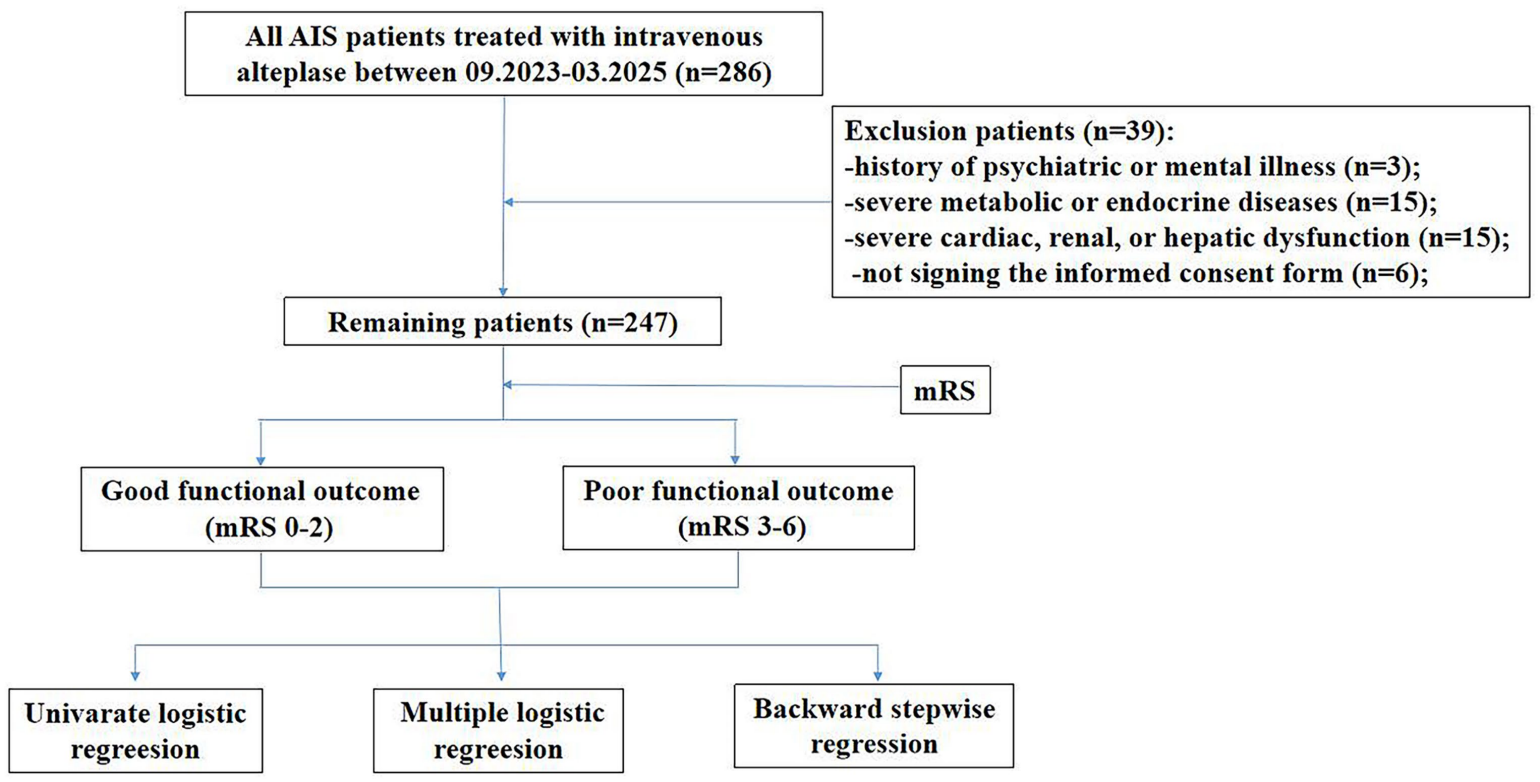
Research flowchart of the study.

This retrospective cohort included 286 AIS patients treated with intravenous alteplase at a tertiary hospital in Chengdu from September 2023 to March 2025. All participants underwent comprehensive lipid and glucose profiling along with mRS assessment. Inclusion criteria: (1) AIS diagnosis per 2014 Chinese Guidelines with CT/MRI confirmation; (2) first-ever stroke; (3) thrombolysis within 4.5 h of onset; (4) age≥18 years. Exclusion criteria: (1) major psychiatric disorders; (2) severe metabolic/endocrine disease; (3) significant heart/kidney/liver dysfunction; (4) lack of informed consent. To ensure the reliability of the results, we removed all unclear or missing data. In strict accordance with the exclusion criteria, we excluded 39 patients. Patients selection methodology appeared in Figure 1. Ethics approval was obtained (Approval No. 2024-S-154) and all participants provided written consent.

### 2.2 Data collection

#### 2.2.1 Clinical baseline data

Clinical data: sex, age, SBP, DBP, smoking (≥1 cigarette/day for ≥1 year), alcohol use (≥100 mL/day, alcohol ≥50%, ≥1 year), history of hypertension, diabetes, dyslipidemia, stroke, coronary heart disease, atrial fibrillation, prior medications (antiplatelets, anticoagulants, antihypertensives, statins, hypoglycemics), pre-thrombolysis the modified Rankin scale (mRS), and door-to-needle time (DNT).

#### 2.2.2 Laboratory data

Laboratory data: platelet count (PLT), international normalized ratio (INR), fibrinogen (Fg), TC: total cholesterol (TC), total triglycerides (TG), high-density lipoprotein cholesterol (HDL), low-density lipoprotein cholesterol (LDL), haemoglobin A1c (HbA1c) and fasting plasma glucose (FPG).

### 2.3 Definition of outcome variables

The mRS scores were assessed by two independent neurosurgeons. Functional outcomes were assessed using the mRS (range 0–6) at baseline, 30 days, and 90 days after thrombolysis. Good outcome was defined as mRS 0–2, poor outcome as mRS 3–6 (19). The definitions of the mRS grades are provided in Supplementary Table S1.

### 2.4 Measurements of exposure variable (TC, TG, LDL, HDL, HbA1c and FPG)

Fasting venous blood (3 mL) were collected on admission day, centrifuged at 3,000 rpm for 15 min, and analyzed using the Mindray BS-2800M automated biochemistry analyzers.

### 2.5 Statistical analysis

Data were processed using R software (version 4.4.3), and “dplyr,” “rms,” “pROC,” and “rmda” were employed. Continuous variables were expressed as mean ± SD or median (IQR) and compared via t-test or Mann–Whitney *U* test. Categorical variables are expressed as number and percentage (*n*, %) and compared using *χ*^2^ test. Significant variables (*p* < 0.05) from univariate analysis were entered into multivariable logistic regression using backward stepwise selection, retaining only variables with *p* < 0.05 in the final model. Model performance was evaluated using ROC (AUC), calibration (1,000 bootstrap resamples), and decision curve analysis (DCA). Odds ratios (OR) with 95% confidence intervals (CI) were calculated, and a *p*-value <0.05 was considered statistically significant.

## 3 Results

### 3.1 Baseline characteristics

A total of 247 AIS patients were analyzed (151 male, 96 female; mean age 70.98 ± 11.53 years). Histories of smoking (44.24%), drinking (44.53%), hypertension (69.23%), diabetes (33.60%), dyslipidemia (25.91%), prior stroke (17.00%), coronary heart disease (17.41%), and atrial fibrillation (24.70%) were recorded. Medication use and lab results were shown in Table 1.

**Table 1:** Overall patient baseline.

Variable	Number of cases (*n* = 247)
Sex	
Male	151 (61.13%)
Female	96 (38.87%)
Age (years)	70.98 ± 11.53
Smoking	
No	107 (43.32%)
Yes	73 (44.24%)
Drinking	
No	137 (55.47%)
Yes	110 (44.53%)
Hypertension	
No	76 (30.77%)
Yes	171 (69.23%)
Diabetes	
No	164 (66.40%)
Yes	83 (33.60%)
Dyslipidemia	
No	183 (74.09%)
Yes	64 (25.91%)
Cerebral infarction	
No	205 (83.00%)
Yes	42 (17.00%)
Coronary heart disease	
No	204 (82.59%)
Yes	43 (17.41%)
Atrial fibrillation	
No	186 (75.30%)
Yes	61 (24.70%)
History of antiplatelet drugs	
No	175 (70.85%)
Yes	72 (29.15%)
History of anticoagulant drugs	
No	226 (91.50%)
Yes	21 (8.50%)
History of antihypertensive drugs	
No	97 (39.27%)
Yes	150 (60.73%)
History of statin drugs	
No	169 (68.42%)
Yes	78 (31.58%)
History of hypoglycemic drugs	
No	185 (74.90%)
Yes	62 (25.10%)
mRS score before thrombolysis	
0	0 (0.00%)
1	19 (7.69%)
2	47 (19.03%)
3	60 (24.29%)
4	100 (40.49%)
5	21 (8.50%)
6	0 (0.00%)
30dmRS	
0	61 (24.70%)
1	62 (25.10%)
2	26 (10.53%)
3	20 (8.10%)
4	32 (12.96%)
5	25 (10.12%)
6	21 (8.50%)
90dmRS	
0	70 (28.34%)
1	59 (23.89%)
2	11 (4.45%)
3	38 (15.38%)
4	23 (9.31%)
5	17 (6.88%)
6	29 (11.74%)
First hospitalization SBP (mmHg)	141.62 ± 21.24
First hospitalization DBP (mmHg)	86.50 ± 18.47
DNT (min)	67.08 ± 19.71
PLT (10^9^/L)	176.78 ± 61.58
INR	1.20 ± 0.32
Fg (g/L)	2.63 ± 0.90
TC (mmol/L)	3.60 ± 0.92
TG (mmol/L)	1.10 ± 0.33
HDL (mmol/L)	1.37 ± 0.37
LDL (mmol/L)	2.23 ± 1.39
HbA1c (%)	7.81 ± 1.56
FPG (mmol/L)	7.29 ± 1.72

Continuous variables were expressed as mean ± SD and categorical variables were expressed as NO. (%). SBP, systolic blood pressure; DBP, diastolic blood pressure; DNT, door to needle time; PLT, platelet; INR, international normalized ratio; Fg, fibrinogen; TC, total cholesterol; TG, total triglycerides; HDL, high-density lipoprotein cholesterol; LDL, low-density lipoprotein cholesterol; HbA1c, haemoglobin A1c; FPG, fasting plasma glucose.

### 3.2 Analysis of different functional outcome groups at 30 days and 90 days after intravenous thrombolysis

At 30 days post-thrombolysis, 149 patients had good outcomes and 98 had poor outcomes. At 90 days, 140 patients were classified as good outcome and 107 as poor outcome. Age, smoking, diabetes, SBP, DBP, DNT, INR, Fg, TC, TG, HDL, LDL, HbA1c and FPG were significantly different between two groups (all *p* < 0.05). Details were shown in Table 2.

**Table 2:** Analysis of different functional outcome groups at 30 days and 90 days.

	30 days			90 days		
Variable	Good functional	Poor functional	*p*	Good functional	Poor functional	*p*
	(mRS 0–2, *N* = 149)	(mRS 3–6, *N* = 98)		(mRS 0–2, *N* = 140)	(mRS 3–6, *N* = 107)	
Age (years)	69.56 ± 11.30	73.13 ± 11.61	0.018	69.47 ± 11.35	72.94 ± 11.53	0.019
Sex			0.239			0.614
Male	96 (64.43%)	55 (56.12%)		88 (62.86%)	63 (58.88%)	
Female	53 (35.57%)	43 (43.88%)		52 (37.14%)	44 (41.12%)	
Smoking			0.009			0.001
No	75 (50.34%)	32 (32.65%)		74 (52.86%)	33 (30.84%)	
Yes	74 (49.66%)	66 (67.35%)		66 (47.14%)	74 (69.16%)	
Drinking			0.073			0.005
No	90 (60.40%)	47 (47.96%)		89 (63.57%)	48 (44.86%)	
Yes	59 (39.60%)	51 (52.04%)		51 (36.43%)	59 (55.14%)	
Hypertension			0.111			0.019
No	52 (34.90%)	24 (24.49%)		52 (37.14%)	24 (22.43%)	
Yes	97 (65.10%)	74 (75.51%)		88 (62.86%)	83 (77.57%)	
Diabetes			0.018			0.009
No	108 (72.48%)	56 (57.14%)		103 (73.57%)	61 (57.01%)	
Yes	41 (27.52%)	42 (42.86%)		37 (26.43%)	46 (42.99%)	
Dyslipidemia			0.975			0.603
No	111 (74.50%)	72 (73.47%)		106 (75.71%)	77 (71.96%)	
Yes	38 (25.50%)	26 (26.53%)		34 (24.29%)	30 (28.04%)	
Cerebral infarction			0.772			0.655
No	125 (83.89%)	80 (81.63%)		118 (84.29%)	87 (81.31%)	
Yes	24 (16.11%)	18 (18.37%)		22 (15.71%)	20 (18.69%)	
Coronary heart disease			0.403			0.099
No	126 (84.56%)	78 (79.59%)		121 (86.43%)	83 (77.57%)	
Yes	23 (15.44%)	20 (20.41%)		19 (13.57%)	24 (22.43%)	
Atrial fibrillation			0.320			0.537
No	116 (77.85%)	70 (71.43%)		108 (77.14%)	78 (72.90%)	
Yes	33 (22.15%)	28 (28.57%)		32 (22.86%)	29 (27.10%)	
History of antiplatelet drugs			0.260			0.350
No	110 (73.83%)	65 (66.33%)		103 (73.57%)	72 (67.29%)	
Yes	39 (26.17%)	33 (33.67%)		37 (26.43%)	35 (32.71%)	
History of anticoagulant drugs			1.000			0.269
No	136 (91.28%)	90 (91.84%)		131 (93.57%)	95 (88.79%)	
Yes	13 (8.72%)	8 (8.16%)		9 (6.43%)	12 (11.21%)	
History of antihypertensive drugs			0.184			0.235
No	64 (42.95%)	33 (33.67%)		60 (42.86%)	37 (34.58%)	
Yes	85 (57.05%)	65 (66.33%)		80 (57.14%)	70 (65.42%)	
History of statin drugs			0.320			0.454
No	106 (71.14%)	63 (64.29%)		99 (70.71%)	70 (65.42%)	
Yes	43 (28.86%)	35 (35.71%)		41 (29.29%)	37 (34.58%)	
History of hypoglycemic drugs			0.384			0.281
No	115 (77.18%)	70 (71.43%)		109 (77.86%)	76 (71.03%)	
Yes	34 (22.82%)	28 (28.57%)		31 (22.14%)	31 (28.97%)	
First hospitalization SBP (mmHg)	137.61 ± 19.52	147.70 ± 22.37	<0.001	137.99 ± 18.69	146.36 ± 23.42	0.003
First hospitalization DBP (mmHg)	84.45 ± 17.65	89.87 ± 19.38	0.036	83.56 ± 15.53	90.60 ± 21.35	0.007
DNT (min)	64.34 ± 16.91	71.24 ± 22.81	0.011	64.31 ± 16.81	70.70 ± 22.53	0.011
PLT (10^9^/L)	174.35 ± 56.39	179.29 ± 66.74	0.585	174.16 ± 57.70	179.26 ± 65.26	0.585
INR	1.24 ± 0.39	1.13 ± 0.17	0.003	1.24 ± 0.40	1.13 ± 0.17	0.003
Fg (g/L)	2.42 ± 0.71	2.96 ± 1.05	<0.001	2.37 ± 0.70	2.98 ± 1.01	<0.001
TC (mmol/L)	3.40 ± 0.93	3.89 ± 0.82	<0.001	3.36 ± 0.89	3.90 ± 0.87	<0.001
TG (mmol/L)	1.05 ± 0.27	1.18 ± 0.40	0.004	1.02 ± 0.26	1.20 ± 0.38	0.004
HDL (mmol/L)	1.42 ± 0.41	1.28 ± 0.30	0.002	1.44 ± 0.41	1.27 ± 0.29	0.002
LDL (mmol/L)	1.85 ± 0.63	2.81 ± 1.93	<0.001	1.81 ± 0.56	2.78 ± 1.88	<0.001
HbA1c (%)	7.63 ± 1.42	8.09 ± 1.72	0.029	7.53 ± 1.36	8.18 ± 1.72	0.029
FPG (mmol/L)	7.00 ± 1.80	7.73 ± 1.48	0.001	6.97 ± 1.79	7.71 ± 1.53	0.001

SBP, systolic blood pressure; DBP, diastolic blood pressure; DNT, door to needle time; PLT, platelet; INR, international normalized ratio; Fg, fibrinogen; TC, total cholesterol; TG, total triglycerides; HDL, high-density lipoprotein cholesterol; LDL, low-density lipoprotein cholesterol; HbA1c, haemoglobin A1c; FPG, fasting plasma glucose.

### 3.3 Risk factors and multiple logistic regression models of 30 days after intravenous thrombolysis

As shown in Table 3, univariate logistic regression analysis showed age, smoking, diabetes, SBP, DBP, DNT, INR, Fg, TC, TG, HDL, LDL, HbA1c and FPG were significant influencing factors (all *p* < 0.05). The results of multivariate logistic regression model was showed in Model 1, and the model parameter table was shown in Supplementary Table S2A. To refine the predictive model, optimized model (Model 2) using backward stepwise regression also showed age (OR: 1.05, 95% CI: 1.02–1.09, *p* = 0.001), smoking (OR: 2.06, 95% CI: 1.02–4.17, *p* = 0.044), diabetes (OR: 2.10, 95% CI: 1.01–4.34, *p* = 0.046), SBP (OR: 1.02, 95% CI: 1.00–1.03, *p* = 0.028), DBP (OR: 1.03, 95% CI: 1.01–1.04, *p* = 0.007), TG (OR: 5.70, 95% CI: 1.89–17.20, *p* = 0.002), HDL (OR: 0.31, 95% CI: 0.12–0.82, *p* = 0.018), LDL (OR: 2.00, 95% CI: 1.33–3.02, *p* = 0.001) and FPG (OR: 1.31, 95% CI: 1.08–1.60, *p* = 0.007) were significant influencing factors of outcome of 30 days after intravenous thrombolysis. The model parameter table of Model 2 was shown in Supplementary Table S2B.

**Table 3:** Analysis of risk factors at 30 days after intravenous thrombolysis.

	Unadjusted		Adjusted to Model 1		Adjusted to Model 2	
Variable	OR (95% CI)	*p*	OR (95% CI)	*p*	OR (95% CI)	*p*
Age (years)	1.03 (1.00, 1.05)	0.018	1.05 (1.01,1.08)	0.005	1.05 (1.02, 1.09)	0.001
Sex						
Male	Reference					
Female	1.41 (0.84, 2.39)	0.194				
Smoking						
No	Reference		Reference			
Yes	2.08 (1.23, 3.57)	0.006	2.08 (1.23, 3.57)	0.048	2.06 (1.02, 4.17)	0.044
Drinking						
No	Reference					
Yes	1.65 (0.99, 2.77)	0.056				
Hypertension						
No	Reference					
Yes	1.65 (0.94, 2.95)	0.084				
Diabetes						
No	Reference		Reference			
Yes	1.97 (1.15, 3.39)	0.014	1.98 (0.94, 4.17)	0.073	2.10 (1.01, 4.34)	0.046
Dyslipidemia						
No	Reference					
Yes	1.06 (0.59, 1.89)	0.855				
Cerebral infarction						
No	Reference					
Yes	1.17 (0.59, 2.30)	0.645				
Coronary heart disease						
No	Reference					
Yes	1.40 (0.72, 2.73)	0.320				
Atrial fibrillation						
No	Reference					
Yes	1.40 (0.78, 2.53)	0.258				
History of antiplatelet drugs						
No	Reference					
Yes	1.43 (0.82, 2.50)	0.210				
History of anticoagulant drugs						
No	Reference					
Yes	0.94 (0.35, 2.34)	0.890				
History of antihypertensive drug						
No	Reference					
Yes	1.48 (0.87, 2.53)	0.147				
History of statin drugs						
No	Reference					
Yes	1.37 (0.79, 2.36)	0.262				
History of hypoglycemic drugs						
No	Reference					
Yes	1.35 (0.75, 2.42)	0.313				
SBP	1.02 (1.01, 1.04)	<0.001	1.02 (1.00, 1.03)	0.053	1.02 (1.00, 1.03)	0.028
DBP	1.02 (1.00, 1.03)	0.035	1.02 (1.00, 1.04)	0.020	1.03 (1.01, 1.04)	0.007
DNT (min)	1.02 (1.00, 1.03)	0.008	1.01 (0.99, 1.03)	0.252		
PLT (10^9^/L)	1.00 (1.00, 1.01)	0.583				
INR	0.18 (0.05, 0.68)	0.011	0.34 (0.06, 1.80)	0.203		
Fg (g/L)	2.05 (1.48, 2.85)	<0.001	1.15 (0.76, 1.76)	0.510		
TC (mmol/L)	1.87 (1.37, 2.55)	<0.001	1.36 (0.87, 2.13)	0.185		
TG (mmol/L)	3.54 (1.55, 8.05)	0.003	4.57 (1.44, 14.44)	0.010	5.70 (1.89, 17.20)	0.002
HDL (mmol/L)	0.33 (0.16, 0.71)	0.004	0.36 (0.13, 1.00)	0.049	0.31 (0.12, 0.82)	0.018
LDL (mmol/L)	2.07 (1.47, 2.91)	<0.001	1.70 (1.15, 2.52)	0.008	2.00 (1.33, 3.02)	0.001
HbA1c (%)	1.21 (1.02, 1.43)	0.025	1.11 (0.90, 1.37)	0.323		
FPG (mmol/L)	1.30 (1.11, 1.52)	0.001	1.22 (0.98, 1.51)	0.072	1.31 (1.08, 1.60)	0.007

SBP, systolic blood pressure; DBP, diastolic blood pressure; DNT, door to needle time; PLT, platelet; INR, international normalized ratio; Fg, fibrinogen; TC, total cholesterol; TG, total triglycerides; HDL, high-density lipoprotein cholesterol; LDL, low-density lipoprotein cholesterol; HbA1c, haemoglobin A1c; FPG, fasting plasma glucose.

### 3.4 Risk factors and multiple logistic regression models of 90 days after intravenous thrombolysis

Multivariate logistic regression model of 90 days was shown in Table 4, and the model parameter table was shown in Supplementary Table S3A. The optimized model showed age (OR: 1.06, 95% CI: 1.02–1.09, *p* = 0.002), smoking (OR: 2.96, 95% CI: 1.40–6.26, *p* = 0.004), diabetes (OR: 3.04, 95% CI: 1.37–6.78, *p* = 0.006), SBP (OR: 1.03, 95% CI: 1.01–1.05, *p* = 0.001). TG (OR: 15.91, 95% CI: 4.24–59.66, *p* < 0.001), HDL (OR: 0.24, 95% CI: 0.08–0.68, *p* = 0.007), LDL (OR: 2.70, 95% CI: 1.58–4.61, *p* < 0.001), HbA1c (OR: 1.32, 95% CI: 1.05–1.66, *p* = 0.018) and FPG (OR: 1.34, 95% CI: 1.08–1.66, *p* = 0.007) were significant influencing factors of outcome of 90 days after intravenous thrombolysis. The model parameter table of Model 2 was shown in Supplementary Table S3B.

**Table 4:** Analysis of risk factors at 90 days after intravenous thrombolysis.

	Unadjusted		Adjusted to Model 1		Adjusted to Model 2	
Variable	OR (95% CI)	*p*	OR (95% CI)	*p*	OR (95% CI)	*p*
Age (years)	1.03 (1.00, 1.05)	0.020	1.05 (1.01, 1.09)	0.008	1.06 (1.02, 1.09)	0.002
Sex						
Male	Reference					
Female	1.18 (0.70, 1.98)	0.528				
Smoking						
No	Reference		Reference			
Yes	2.50 (1.48, 4.28)	0.001	2.86 (0.97, 8.44)	0.057	2.96 (1.40, 6.26)	0.004
Drinking						
No	Reference		Reference			
Yes	2.14 (1.28, 3.59)	0.004	1.00 (0.35, 2.90)	0.997		
Hypertension						
No	Reference		Reference			
Yes	2.03 (1.16, 3.64)	0.013	1.11 (0.43, 2.87)	0.826		
Diabetes						
No	Reference		Reference			
Yes	2.09 (1.22, 3.60)	0.007	3.01 (1.23, 7.36)	0.016	3.04 (1.37, 6.78)	0.006
Dyslipidemia						
No	Reference					
Yes	1.21 (0.68, 2.16)	0.508				
Cerebral infarction						
No	Reference					
Yes	1.23 (0.63, 2.41)	0.541				
Coronary heart disease						
No	Reference					
Yes	1.83 (0.94, 3.61)	0.074				
Atrial fibrillation						
No	Reference					
Yes	1.25 (0.70, 2.25)	0.448				
History of antiplatelet drugs						
No	Reference					
Yes	1.35 (0.78, 2.35)	0.287				
History of anticoagulant drugs						
No	Reference					
Yes	1.83 (0.74, 4.70)	0.194				
History of antihypertensive drug						
No	Reference					
Yes	1.42 (0.84, 2.40)	0.190				
History of statin drugs						
No	Reference					
Yes	1.28 (0.74, 2.19)	0.379				
History of hypoglycemic drugs						
No	Reference					
Yes	1.43 (0.80, 2.56)	0.225				
SBP	1.02 (1.01, 1.03)	0.003	1.01 (0.99, 1.03)	0.262		
DBP	1.02 (1.01, 1.04)	0.006	1.03 (1.01, 1.06)	0.003	1.03 (1.01, 1.05)	0.001
DNT (min)	1.02 (1.00, 1.03)	0.013	1.01 (0.98, 1.03)	0.651		
PLT (10^9^/L)	1.00 (1.00, 1.01)	0.571				
INR	0.18 (0.05, 0.66)	0.009	0.38 (0.08, 1.90)	0.241		
Fg (g/L)	2.43 (1.71, 3.47)	<0.001	1.47 (0.91, 2.40)	0.119		
TC (mmol/L)	2.04 (1.48, 2.80)	<0.001	1.62 (0.97, 2.71)	0.065		
TG (mmol/L)	6.27 (2.58, 15.22)	<0.001	16.16 (4.01, 65.17)	<0.001	15.91 (4.24, 59.66)	<0.001
HDL (mmol/L)	0.28 (0.13, 0.59)	<0.001	0.27 (0.09, 0.85)	0.025	0.24 (0.08, 0.68)	0.007
LDL (mmol/L)	2.30 (1.58, 3.35)	<0.001	1.85 (1.14, 3.01)	0.013	2.70 (1.58, 4.61)	<0.001
HbA1c (%)	1.32 (1.11, 1.57)	0.001	1.32 (1.03, 1.68)	0.027	1.32 (1.05, 1.66)	0.018
FPG (mmol/L)	1.30 (1.11, 1.52)	0.001	1.26 (1.00, 1.60)	0.051	1.34 (1.08, 1.66)	0.007

SBP, systolic blood pressure; DBP, diastolic blood pressure; DNT, door to needle time; PLT, platelet; INR, international normalized ratio; Fg, fibrinogen; TC, total cholesterol; TG, total triglycerides; HDL, high-density lipoprotein cholesterol; LDL, low-density lipoprotein cholesterol; HbA1c, haemoglobin A1c; FPG, fasting plasma glucose.

### 3.5 Nomogram for predicting prognosis after intravenous thrombolysis

**Figure 2:**
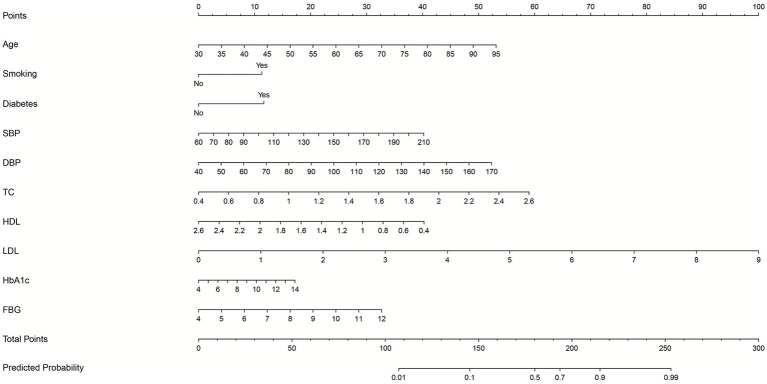
The nomogram for predicting prognosis after intravenous thrombolysis. (Scores were obtained from each scale according to patient-specific indicators, with total scores corresponding to predicted probabilities after summation. Higher total scores indicated greater poor functional outcome risk).

This research developed a nomogram by integrating factors assessed at both 30 day and 90 day time points (Figure 2). A VIF value more than 10 or a TOL less than 0.1 indicate multicollinearity. The final model demonstrated no significant multicollinearity among the variables (Supplementary Table S4). The result showed higher age, smoking, diabetes, elevated SBP/DBP, increased TC, low HDL, elevated LDL, high HbA1c, and high FPG were risk factors of prognosis after intravenous thrombolysis. When using the nomogram to predict prognosis after intravenous thrombolysis in stroke patients, doctors can place on the corresponding axes based on the patient’s risk factors. The assigned scores from each factor were summed to obtain a total score. A higher total score indicated a greater risk of poor functional outcome. For management, it can aid in rapidly stratifying risk and prioritizing monitoring or intervention for high risk patients.

### 3.6 Evaluation of nomogram for predicting prognosis after intravenous thrombolysis

**Figure 3:**
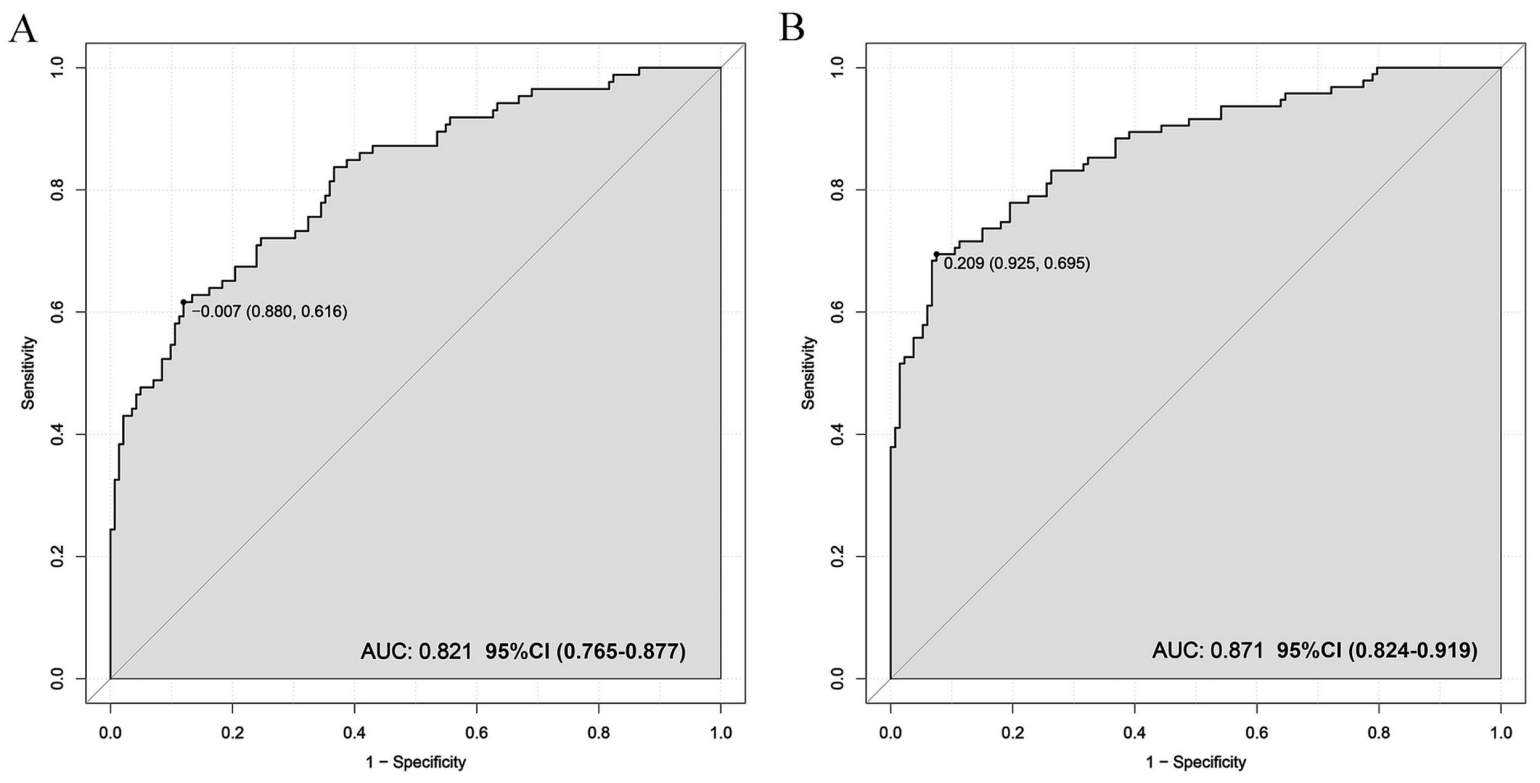
ROC curve of the nomogram for predicting prognosis after intravenous thrombolysis. **(A)** 30 days; **(B)** 90 days. (30 days nomogram’s AUC was 0.821, 95% CI: 0.765–0.877; 90 days nomogram’s AUC was 0.871, 95% CI: 0.824–0.919. AUC over 0.7 indicating that the model possesses a relatively good discriminatory ability and accuracy).

**Figure 4:**
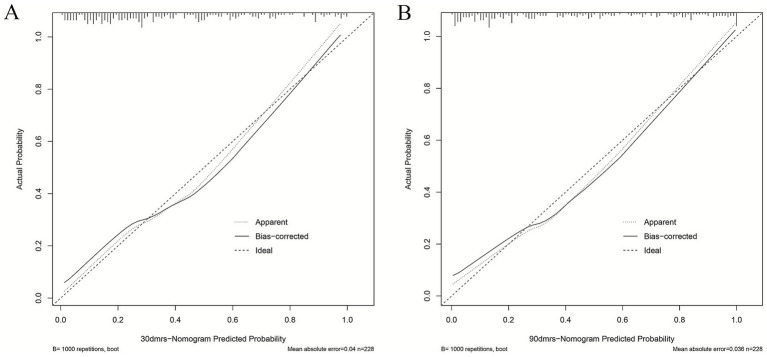
The calibration curve of the nomogram for predicting prognosis after intravenous thrombolysis. **(A)** 30 days; **(B)** 90 days. (The calibration curve was constructed using 1,000 bootstrap repetitions. The diagonal dashed line represents the ideal case of perfect prediction, while the solid line indicates the actual performance of our model. Closer agreement between the two lines signifies better predictive accuracy).

**Figure 5:**
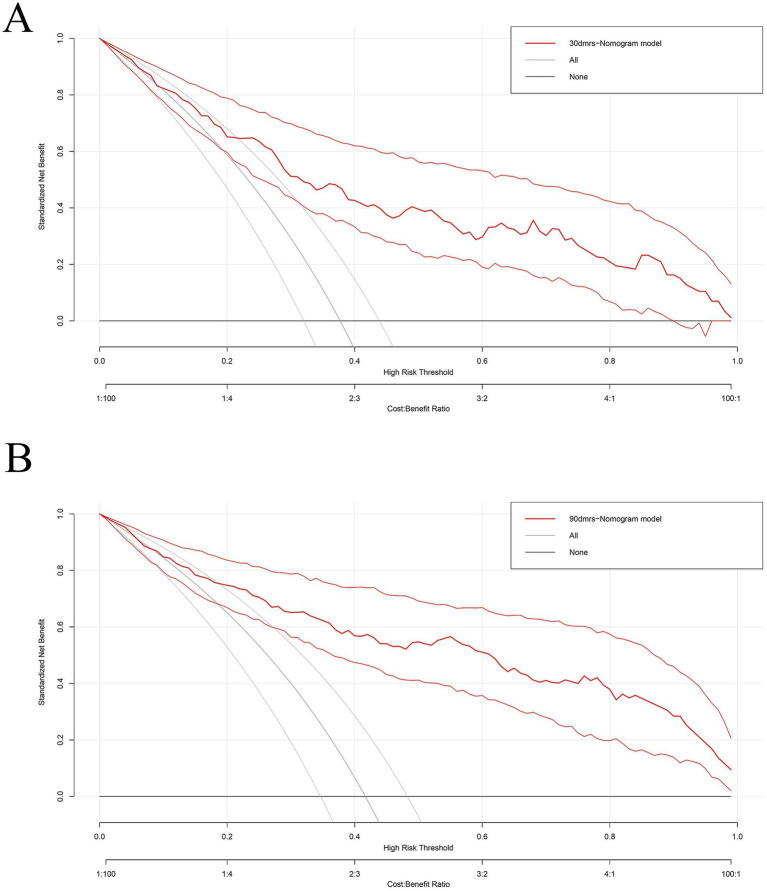
The decision curve of the nomogram for predicting prognosis after intravenous thrombolysis. **(A)** 30 days; **(B)** 90 days. (Nomograms offered a greater net benefit compared to both the “treat-all” and “treat-none” approaches over a threshold probability range from 5 to 100%, confirming its clinical utility in routine practice).

We evaluated this nomogram at two distinct time points, the nomogram achieved a strong discriminatory ability. Hosmer–Lemeshow goodness-of-fit test showed 30 days nomogram’s *χ*^2^ = 7.939, *p* = 0.439; 90 days nomogram’s *χ*^2^ = 6.554, *p* = 0.5851. At 30 days after intravenous thrombolysis, the AUC of 0.821 (95% CI: 0.765–0.877) (Figure 3A), the optimal cutoff value was −0.007, with the maximum Youden index of 0.496, yielding a sensitivity of 88.0% and a specificity of 61.6% for prediction; At 90 days after intravenous thrombolysis, the AUC of 0.871 (95% CI: 0.824–0.919) (Figure 3B), the optimal cutoff value was 0.209, with the maximum Youden index of 0.620, yielding a sensitivity of 92.5% and a specificity of 69.5% for prediction. This study employed bootstrap calibration curves (1,000 replicates) to quantify prediction-actuality deviations. The solid line indicates model outputs, contrasting with the ideal-fit diagonal dashed line. Closer convergence between these lines indicated enhanced predictive accuracy. As shown in Figure 4, the absolute error between the simulated and actual curves were all 0.04, indicating good agreement between predicted and observed outcomes. DCA demonstrated the nomogram’s outperformance of both “treat-all” and “treat-none” approaches throughout the threshold probability spectrum (5–100%), justifying its clinical utility (Figure 5).

### 3.7 Linear association between predictive factors and prognosis after intravenous thrombolysis

We used restricted cubic spline (RCS) analysis to better demonstrate the relationship between predictive factors (TC, HDL, LDL, HbA1c, FBG) and prognosis at 30 days (Supplementary Figures S1A–E) and at 90 days (Supplementary Figures S2A–E), and observed significant linear correlations between the predictors and poor functional outcome. We conducted threshold effect analysis and found inflection points (TC was 1.05, HDL was 1.29, LDL was 1.96, HbA1c was 7.70, FBG was 7.22). Observations indicated that when TC, LDL, HbA1c, FBG was below the inflection point, the risk of poor functional outcome was lower, when TC, LDL, HbA1c, FBG exceeded the inflection point, the risk increases rapidly. However, poor functional outcome risk remained high below this threshold but reduced significantly when HDL exceeded 1.29.

## 4 Discussion

Alteplase intravenous thrombolysis is the cornerstone of ultra-early treatment for AIS, significantly improving patient outcomes by rapidly restoring blood flow (20, 21). However, approximately 20–40% of patients still experience poor outcomes despite receiving thrombolytic therapy, including worsening neurological deficits or death, as their prognosis is influenced by multiple factors (22). This study identified glucose metabolism abnormalities, lipid levels, age, smoking, and blood pressure as independent risk factors affecting prognosis, a finding that closely aligns with conclusions from previous research.

### 4.1 Glucose metabolism (FPG, HbA1c, diabetes) affects the prognosis of thrombolytic therapy in stroke patients

Vakilipour et al. (23) found that the mean levels of FPG were higher than normal in stroke patients. Ding et al. (24) found that FPG level was significantly higher in the poor functional outcome group (mRS ≥2) compared to the favorable outcome group (6.33 vs. 5.10 mmol/L, *p* = 0.001). These result further confirmed that abnormal glucose metabolism was an independent risk factor. The relationship between glucose metabolism and IS involves several potential mechanisms: (1) Stress hyperglycemia: Abnormal neuroendocrine regulatory mechanisms trigger a stress response, leading to increased hepatic glucose production and/or insulin resistance. Furthermore, a history of chronic hyperglycemia/diabetes may reduce the body’s sensitivity to the neuroendocrine system, thereby exerting an additional influence on the relationship between blood glucose and stroke outcomes (25, 26). (2) Exacerbation of ischemic injury: Hyperglycemia activates the NLRP3 inflammasome, promoting the release of pro-inflammatory cytokines such as IL-1β and TNF-α, which aggravates oxidative stress and inflammation-related responses, thereby worsening neuronal death in the ischemic penumbra (27, 28). (3) Blood–brain barrier disruption: Hyperglycemia leads to disruption of the blood–brain barrier, further increasing the risk of cerebral edema (29).

Furthermore, previous studies have found HbA1c was associated with poor outcomes after thrombolytic therapy in stroke patients (30, 31). HbA1c is the product formed by the binding of haemoglobin in red blood cells to glucose in the blood. HbA1c modulates oxidative reactions in the body. Excessively high levels of HbA1c reduce the conversion rate of oxyhemoglobin, impair haemoglobin’s oxygen-carrying capacity, and lead to histotoxic hypoxia (32–34).

### 4.2 Dyslipidemia (LDL, HDL, TG) affects the prognosis of thrombolytic therapy in stroke patients

Amarenco et al. (35) and Xu et al. (36) also found LDL levels were associated with prognosis of stroke patients. Rajabpour et al. (37) also found an increase in LDL-c levels and a decrease in HDL-c levels in stroke patients. LDL exacerbates neurological deterioration after thrombolysis by promoting the rupture of atherosclerotic plaques and activating the inflammasome, such as interleukin-1β (IL-1β) and interleukin-6 (IL-6) (5). HDL influences outcomes after intravenous alteplase thrombolysis through multiple mechanisms. Studies indicate that HDL enhances blood–brain barrier (BBB) integrity via the SR-BI receptor-dependent pathway, reducing the risk of hemorrhagic transformation post-thrombolysis (38). Furthermore, HDL improves neurological recovery after thrombolysis by promoting cholesterol reverse transport and exerting anti-inflammatory effects. However, this protective effect is attenuated in patients with comorbid diabetes or chronic kidney disease (39). TG significantly increases the risk of poor outcomes in IS patients through multiple pathways (40, 41): it promotes atherosclerotic plaque formation, increases blood viscosity, triggers inflammatory responses, and induces endothelial cell apoptosis by activating the NLRP3 inflammasome; it also inhibits fibrinolytic system function, thereby predisposing to thrombosis (42).

### 4.3 Age affects the prognosis of thrombolytic therapy in stroke patients

A Chinese study demonstrated that patients over 80 years old who received thrombolysis had a 12.6% higher proportion achieving an mRS score of 0–2 at 90 days compared to the non-thrombolysis group, but also carried a 3.22-fold increased risk of symptomatic intracranial hemorrhage (sICH) (43). This aligns with European and American studies confirming higher sICH incidence after thrombolysis in elderly patients (22). This may be attributed to: (1) Reperfusion efficiency: Younger patients exhibit superior collateral circulation, resulting in significantly higher recanalization rates following thrombolysis (18% improvement vs. elderly patients; *p* = 0.003) (44). (2) Biological vulnerabilities: Advanced age is associated with microbleeds and leukoaraiosis, pathologies that compromise blood–brain barrier integrity and heighten susceptibility to hemorrhage.

### 4.4 Smoking affects the prognosis of thrombolytic therapy in stroke patients

Smoking was associated with poor functional outcomes in ischemic stroke patients receiving alteplase thrombolysis (45). Liu et al. (46) found that smokers had an 80% higher risk of an mRS score >2 at 90 days compared to non-smokers. Smoking induces systemic inflammation by mobilizing neutrophils and monocytes, thereby exacerbating post-ischemic neuroinflammation and promoting infarct expansion (47). Moreover, animal experiments showed that: chronic nicotine exposure (simulated smoking) reduced hematoma expansion after spontaneous sICH through red blood cell-derived particles (RMPs) (26% reduction in males and 31% reduction in females), and the effect was sustained for 4.5 h after sICH (48).

### 4.5 Blood pressure affects the prognosis of thrombolytic therapy in stroke patients

Retrospective analyses have demonstrated a graded relationship between systolic blood pressure and the likelihood of hemorrhagic transformation, with SBP values exceeding 170 mmHg conferring an approximately fourfold increase in risk relative to the 141–150 mmHg reference range (49). A 2021 meta-analysis of 5,874 patients identified a mean 24-h SBP >160 mmHg following endovascular thrombectomy (EVT) as an independent risk predictor of poorer outcomes (50). Mechanistically, (1) acute increased blood pressure exacerbates cerebrovascular dysregulation, thereby promoting cerebral edema and hemorrhagic transformation (51); (2) acute blood pressure fluctuations compromise cerebral perfusion within the ischemic penumbra (52), while after stroke, vascular regulation ability is impaired, and systemic blood pressure further affects CBF within the penumbra (53).

However, this study had the following limitations: (1) Single-center retrospective design: The sample was derived from a single institution’s clinical database, which may introduce selection bias (e.g., regional, ethnic, or treatment protocol variations). Furthermore, the retrospective design limits comprehensive control of confounding factors (e.g., dynamic changes in patient lifestyles and comorbidities), potentially affecting the accuracy of prognostic factor analysis. These factors restricted the generalisability of the findings, and external validation in multi-centre prospective cohorts is essential before any clinical application. (2) Limited sample size: The inclusion of 284 patients represents a relatively small cohort, which may reduce statistical power. (3) Limited factors: This study only incorporated routine clinical indicators (e.g., glucose metabolism, lipid profiles, age) while omitting inflammatory markers (e.g., IL-6, TNF-α), genetic variants (e.g., NINJ2 gene), and neuroimaging features (e.g., infarct core volume); (4) No stratified analysis was performed: the original data did not included TOAST criteria or details on vascular occlusion, making it impossible to investigate whether differences exist based on etiology. To address these limitations, future studies should focus on the following: (1) Prospective multicenter investigations: Expand sample sizes and establish multicenter cohorts to validate the reliability of current findings. (2) Stratified analysis: Based on the TOAST criteria or the occluded vessel, to investigate whether glucose-lipid effects differ across etiologies. (3) Mechanistic exploration: Conduct basic science experiments to elucidate the molecular mechanisms through which dysregulated glucose/lipid metabolism affects reperfusion efficacy, and identify potential therapeutic targets. (4) Personalized intervention strategies: Develop tiered thrombolysis protocols (e.g., combined antiplatelet therapy or endovascular interventions) based on risk stratification models, and establish precision treatment pathways for high-risk populations.

## 5 Conclusion

This study developed and validated a predictive model based on glucose and lipid profiles using clinical data from 247 stroke patients who underwent intravenous thrombolysis. The model demonstrated strong and consistent predictive performance at both 30 and 90 days, confirming its reliability and clinical utility. By identifying key metabolic and vascular risk factors, this tool enables clinicians to recognize patients at high risk of poor outcomes and supports risk-stratified management strategies. Future studies involving external validation are warranted to confirm its generalizability across diverse populations and clinical settings.

## Data Availability

The original contributions presented in the study are included in the article/Supplementary material, further inquiries can be directed to the corresponding author.

## References

[1] GBD 2021 Stroke Risk Factor Collaborators. Global, regional, and national burden of stroke and its risk factors, 1990–2021: a systematic analysis for the Global Burden of Disease Study 2021. Lancet Neurol. (2024) 23:973–1003. doi: 10.1016/S1474-4422(24)00369-7 39304265 PMC12254192

[2] Reina SA Llabre MM Allison MA Wilkins JT Mendez AJ Arnan MK . HDL cholesterol and stroke risk: the multi-ethnic study of atherosclerosis. Atherosclerosis. (2015) 243:314–9. doi: 10.1016/j.atherosclerosis.2015.09.031, 26425994 PMC4609625

[3] Oliveira LC Ponciano A Tuozzo C Viswanathan A Rost NS Etherton MR. Poststroke disability: association between sex and patient-reported outcomes. Stroke. (2023) 54:345–53. doi: 10.1161/STROKEAHA.122.041502, 36689580

[4] Dai L Xu J Zhang Y Wang A Chen Z Mo J . Cumulative burden of lipid profiles predict future incidence of ischaemic stroke and residual risk. Stroke Vasc Neurol. (2021) 6:581–8. doi: 10.1136/svn-2020-000726, 33827914 PMC8717800

[5] Verma S Mazer CD Connelly KA. Inflammation and cholesterol at the crossroads of vascular risk. Cell Metab. (2023) 35:1095–8. doi: 10.1016/j.cmet.2023.06.011, 37437543

[6] Freiberg JJ Tybjaerg-Hansen A Jensen JS Nordestgaard BG. Nonfasting triglycerides and risk of ischemic stroke in the general population. JAMA. (2008) 300:2142–52. doi: 10.1001/jama.2008.621, 19001625

[7] Wang Y Jing J Meng X Pan Y Wang Y Zhao X . The Third China National Stroke Registry (CNSR-III) for patients with acute ischaemic stroke or transient ischaemic attack: design, rationale and baseline patient characteristics. Stroke Vasc Neurol. (2019) 4:158–64. doi: 10.1136/svn-2019-000242, 31709123 PMC6812638

[8] Niu H Wang Y Yang N Chu M Mao X Wang D . Elevated remnant cholesterol improves the prognosis of patients with ischemic stroke and malnutrition: a cohort-based study. Stroke. (2025) 56:2057–67. doi: 10.1161/STROKEAHA.124.048785, 40326332

[9] Xu J Zhang X Jin A Pan Y Li Z Meng X . Trends and risk factors associated with stroke recurrence in China, 2007–2018. JAMA Netw Open. (2022) 5:e2216341. doi: 10.1001/jamanetworkopen.2022.16341, 35704318 PMC9201671

[10] Fox KM Tai MH Kostev K Hatz M Qian Y Laufs U. Treatment patterns and low-density lipoprotein cholesterol (LDL) goal attainment among patients receiving high- or moderate-intensity statins. Clin Res Cardiol. (2018) 107:380–8. doi: 10.1007/s00392-017-1193-z, 29273856 PMC5913378

[11] Nicholls SJ. PCSK9 inhibitors and reduction in cardiovascular events: current evidence and future perspectives. Kardiol Pol. (2023) 81:115–22. doi: 10.33963/KP.a2023.0030, 36739653

[12] Bogenschutz KM Fisher DS Wright GW. Acute ischemic stroke: a guideline-based overview of evaluation and management. JAAPA. (2025) 38:13–20. doi: 10.1097/01.JAA.0000000000000203, 40197996

[13] Schwartz GG Steg PG Szarek M Bhatt DL Bittner VA Diaz R . Alirocumab and cardiovascular outcomes after acute coronary syndrome. N Engl J Med. (2018) 379:2097–107. doi: 10.1056/NEJMoa1801174, 30403574

[14] Wang X Li X Xu Y Li R Yang Q Zhao Y . Effectiveness of intravenous r-tPA versus UK for acute ischaemic stroke: a nationwide prospective Chinese registry study. Stroke Vasc Neurol. (2021) 6:603–9. doi: 10.1136/svn-2020-000640, 33903179 PMC8717806

[15] Adusumilli G Pederson JM Hardy N Kallmes KM Hutchison K Kobeissi H . Mechanical thrombectomy in anterior vs. posterior circulation stroke: a systematic review and meta-analysis. Interv Neuroradiol. (2024) 30:307–16. doi: 10.1177/15910199221100796, 35549748 PMC11310733

[16] Chen HS Cui Y Zhou ZH Dai YJ Li GH Peng ZL . Effect of argatroban plus intravenous alteplase vs intravenous alteplase alone on neurologic function in patients with acute ischemic stroke: the ARAIS randomized clinical trial. JAMA. (2023) 329:640–50. doi: 10.1001/jama.2023.0550, 36757755 PMC9912168

[17] Yan S Zhou Y Lansberg MG Liebeskind DS Yuan C Yu H . Alteplase for posterior circulation ischemic stroke at 4.5 to 24 hours. N Engl J Med. (2025) 392:1288–96. doi: 10.1056/NEJMoa2413344, 40174223

[18] Hu Y Wu S Zhang H Wang K Zhang L Ma Y . Correction: efficacy and safety of various intravenous thrombolytics for acute ischemic stroke (AIS) at various dosages: a systematic review and network meta-analysis. Neurol Ther. (2025) 14:525. doi: 10.1007/s40120-024-00709-3, 39812737 PMC11906948

[19] Powers WJ Rabinstein AA Ackerson T Adeoye OM Bambakidis NC Becker K . Guidelines for the early management of patients with acute ischemic stroke: 2019 update to the 2018 guidelines for the early management of acute ischemic stroke: a guideline for healthcare professionals from the American Heart Association/American Stroke Association. Stroke. (2019) 50:e344–418. doi: 10.1161/STR.0000000000000211, 31662037

[20] National Institute of Neurological Disorders and Stroke rt-PA Stroke Study Group. Tissue plasminogen activator for acute ischemic stroke. N Engl J Med. (1995) 333:1581–7. doi: 10.1056/NEJM199512143332401 7477192

[21] Ma H Campbell BCV Parsons MW Churilov L Levi CR Hsu C . Thrombolysis guided by perfusion imaging up to 9 hours after onset of stroke. N Engl J Med. (2019) 380:1795–803. doi: 10.1056/NEJMoa1813046, 31067369

[22] Sandercock P Wardlaw JM Lindley RI Dennis M Cohen G Innes K . The benefits and harms of intravenous thrombolysis with recombinant tissue plasminogen activator within 6 h of acute ischaemic stroke (the third international stroke trial [IST-3]): a randomised controlled trial. Lancet. (2012) 379:2352–63. doi: 10.1016/S0140-6736(12)60768-5 22632908 PMC3386495

[23] Vakilipour P Ala A Sanaie S Panahzadeh Z Ghafouri RR Vahdati SS. Evaluation of nutritional status of patients referred with stroke. J Exp Clin Med. (2024) 41:346–50. doi: 10.52142/omujecm.41.2.22

[24] Ding ZY Li GS Zhao XQ. Relationship between high levels of fasting blood glucose and functional outcome after intravenous thrombolysis in patients with ischemic stroke. Chin J Stroke. (2024) 19:293–8. doi: 10.3969/j.issn.1673-5765.2024.03.007

[25] Southerland AM Mayer SA Chiota-McCollum NA Bolte AC Pauls Q Pettigrew LC . Glucose control and risk of symptomatic intracerebral hemorrhage following thrombolysis for acute ischemic stroke: a SHINE trial analysis. Neurology. (2024) 102:e209323. doi: 10.1212/WNL.0000000000209323, 38626363 PMC11175634

[26] Dungan KM Braithwaite SS Preiser JC. Stress hyperglycaemia. Lancet. (2009) 373:1798–807. doi: 10.1016/S0140-6736(09)60553-5, 19465235 PMC3144755

[27] Luitse MJ Biessels GJ Rutten GE Kappelle LJ. Diabetes, hyperglycaemia, and acute ischaemic stroke. Lancet Neurol. (2012) 11:261–71. doi: 10.1016/S1474-4422(12)70005-4, 22341034

[28] Koracevic GP. Proposal of a new approach to study and categorize stress hyperglycemia in acute myocardial infarction. J Emerg Med. (2016) 51:31–6. doi: 10.1016/j.jemermed.2015.03.047, 27041491

[29] Dietrich WD Alonso O Busto R. Moderate hyperglycemia worsens acute blood-brain barrier injury after forebrain ischemia in rats. Stroke. (1993) 24:111–6. doi: 10.1161/01.str.24.1.111, 8418533

[30] Wang MD Teng RX Ma RR Yang DC Qin L. Study of glycosylated hemoglobin inpredicting prognosis in patients with cerebral infarction and rt-PA intravenous thrombolysis. Int J Lab Med. (2022) 43:424–7. doi: 10.3969/j.issn.1673-4130.2022.04.009

[31] Yue F Wang Z Pu J Zhang M Liu Y Han H . HbA1c and clinical outcomes after endovascular treatment in patients with posterior circulation large vessel occlusion: a subgroup analysis of a nationwide registry (BASILAR). Ther Adv Neurol Disord. (2020) 13:1756286420981354. doi: 10.1177/1756286420981354, 33447263 PMC7780201

[32] Sun W Song HQ Shen HX Wu X He XN Huang XQ. Effects of glycated hemoglobin and random blood glucose on the prognosis of patients with acute large-vessel occlusive ischemic stroke treated with endovascular therapy. Chin J Cerebrovasc Dis. (2023) 20:316–24. doi: 10.3969/j.issn.1672-5921.2023.05.004

[33] Alhawiti NM Elsokkary EM Aldali JA Alotaibi BA. Investigating the impact of glycated hemoglobin levels on stroke severity in patients with acute ischemic stroke. Sci Rep. (2025) 15:12114. doi: 10.1038/s41598-025-95305-2, 40204797 PMC11982240

[34] Hjalmarsson C Manhem K Bokemark L Andersson B. The role of prestroke glycemic control on severity and outcome of acute ischemic stroke. Stroke Res Treat. (2014) 2014:694569. doi: 10.1155/2014/694569, 25295219 PMC4175748

[35] Amarenco P Bogousslavsky J Callahan A 3rd Goldstein LB Hennerici M Rudolph AE . High-dose atorvastatin after stroke or transient ischemic attack. N Engl J Med. (2006) 355:549–59. doi: 10.1056/NEJMoa061894 16899775

[36] Xu J Chen Z Wang M Mo J Jing J Yalkun G . Low LDL-C level and intracranial haemorrhage risk after ischaemic stroke: a prospective cohort study. Stroke Vasc Neurol. (2023) 8:127–33. doi: 10.1136/svn-2022-001612, 36162902 PMC10176994

[37] Rajabpour M Ala A Sadeghi-Hokmabadi E Amiri H Rostamnezhad S Vandati S. Investigating the relationship between lipid profiles of stroke patients at the time of admission and their outcome. OBM Neurobiol. (2024) 8:233. doi: 10.21926/obm.neurobiol.2403233

[38] Tran-Dinh A Levoye A Couret D Galle-Treger L Moreau M Delbosc S . High-density lipoprotein therapy in stroke: evaluation of endothelial SR-BI-dependent neuroprotective effects. Int J Mol Sci. (2020) 22:106. doi: 10.3390/ijms22010106, 33374266 PMC7796353

[39] Xiao Y Yu B Chao C Wang S Hu D Wu C . Chinese expert consensus on blood lipid management in patients with diabetes (2024 edition). J Transl Intern Med. (2024) 12:325–43. doi: 10.2478/jtim-2024-0014, 39360162 PMC11444477

[40] Hoshino T Ishizuka K Toi S Mizuno T Nishimura A Wako S . Prognostic role of hypertriglyceridemia in patients with stroke of atherothrombotic origin. Neurology. (2022) 98:e1660–9. doi: 10.1212/WNL.0000000000200112, 35296551

[41] Annemans L Stock JK Chapman MJ. PCSK9 inhibition, atherosclerotic cardiovascular disease, and health economics: challenges at the crossroads. J Clin Lipidol. (2019) 13:714–20. doi: 10.1016/j.jacl.2019.07.005, 31427270

[42] Shi H Kokoeva MV Inouye K Tzameli I Yin H Flier JS. TLR4 links innate immunity and fatty acid-induced insulin resistance. J Clin Invest. (2006) 116:3015–25. doi: 10.1172/JCI28898, 17053832 PMC1616196

[43] Li C Jiang Y Gu HQ Wang M Chen Z Yang X . Characteristics, temporal trends and outcomes of intravenous thrombolysis in Chinese patients aged >80 years who had a stroke. Stroke Vasc Neurol. (2025) 10:431–40. doi: 10.1136/svn-2024-003427, 39481878 PMC12415639

[44] Emberson J Lees KR Lyden P Blackwell L Albers G Bluhmki E . Effect of treatment delay, age, and stroke severity on the effects of intravenous thrombolysis with alteplase for acute ischaemic stroke: a meta-analysis of individual patient data from randomised trials. Lancet. (2014) 384:1929–35. doi: 10.1016/S0140-6736(14)60584-5, 25106063 PMC4441266

[45] Schlemm L Kufner A Boutitie F Nave AH Gerloff C Thomalla G . Current smoking does not modify the treatment effect of intravenous thrombolysis in acute ischemic stroke patients-a post-hoc analysis of the WAKE-UP trial. Front Neurol. (2019) 10:1239. doi: 10.3389/fneur.2019.01239, 31824412 PMC6883001

[46] Liu SY Cao WF Wu LF Xiang ZB Liu SM Liu HY . Effect of glycated hemoglobin index and mean arterial pressure on acute ischemic stroke prognosis after intravenous thrombolysis with recombinant tissue plasminogen activator. Medicine. (2018) 97:e13216. doi: 10.1097/MD.0000000000013216, 30544380 PMC6310570

[47] Li H Li X Gao S Wang D Gao X Li Y . Exposure to cigarette smoke augments post-ischemic brain injury and inflammation via mobilization of neutrophils and monocytes. Front Immunol. (2019) 10:2576. doi: 10.3389/fimmu.2019.02576, 31787973 PMC6853894

[48] Rehni AK Cho S Zhang Z Khushal P Raval AP Koch S . Red cell microparticles suppress hematoma growth following intracerebral hemorrhage in chronic nicotine-exposed rats. Int J Mol Sci. (2022) 23:15167. doi: 10.3390/ijms232315167, 36499494 PMC9736308

[49] Ahmed N Wahlgren N Brainin M Castillo J Ford GA Kaste M . Relationship of blood pressure, antihypertensive therapy, and outcome in ischemic stroke treated with intravenous thrombolysis: retrospective analysis from Safe Implementation of Thrombolysis in Stroke-International Stroke Thrombolysis Register (SITS-ISTR). Stroke. (2009) 40:2442–9. doi: 10.1161/STROKEAHA.109.548602, 19461022

[50] Katsanos AH Malhotra K Ahmed N Seitidis G Mistry EA Mavridis D . Blood pressure after endovascular thrombectomy and outcomes in patients with acute ischemic stroke: an individual patient data meta-analysis. Neurology. (2022) 98:e291–301. doi: 10.1212/WNL.0000000000013049, 34772799

[51] Jang Y Zhang YW Wu T Deng BQ. Relationship between blood pressure variability and prognosis in acute ischemic stroke patients receiving intravenous thrombolysis. Acad J Second Mil Univ. (2016) 37:1201–5. doi: 10.16781/j.0258-879x.2016.10.1201

[52] Gill D Cox T Aravind A Wilding P Korompoki E Veltkamp R . A fall in systolic blood pressure 24 hours after thrombolysis for acute ischemic stroke is associated with early neurological recovery. J Stroke Cerebrovasc Dis. (2016) 25:1539–43. doi: 10.1016/j.jstrokecerebrovasdis.2016.03.002, 27053029

[53] Jordan JD Powers WJ. Cerebral autoregulation and acute ischemic stroke. Am J Hypertens. (2012) 25:946–50. doi: 10.1038/ajh.2012.53 22573015

