# Supplementary material for: Associations between blood glucose-lipid levels and post-intravenous thrombolysis outcomes in stroke patients: a retrospective study utilizing logistic regression analysis
Source: Front Neurol. 2026 Feb 18;17:1701394. doi: 10.3389/fneur.2026.1701394 (PMC12956658; doi:10.3389/fneur.2026.1701394)
Supplement: Supplementary file 1 [file Table_1.docx]

**Supplementary Materials**

**Table S1: Grading Criteria of the mRS**

| **Parameter** | **Criteria** |
| --- | --- |
| Grade 0 | No symptoms. |
| Grade 1 | Symptoms present but no significant disability; able to carry out all usual activities. |
| Grade 2 | Moderate disability; unable to perform all previous activities but able to attend to self-care without assistance. |
| Grade 3 | Moderate disability requiring some help, though able to walk without support. |
| Grade 4 | Severe disability; unable to walk independently and unable to manage self-care unassisted. |
| Grade 5 | Severe disability; bedridden, incontinent, and requiring constant care. |
| Grade 6 | Death. |

mRS:Modified Rankin Scale

**Table S2A Model parameter table in multiple logistic regression model of 30 days after intravenous thrombolysis**

| **Variable** | ***β*** | ***S.D*** | Wald χ² | ***OR(95%CI)*** | ***P-value*** |
| --- | --- | --- | --- | --- | --- |
| Intercept | -13.594 | 3.095 | 19.292 | - | <0.001 |
| Age | 0.047 | 0.017 | 7.904 | 1.048(1.014-1.082) | 0.005 |
| Smoking (yes) | 0.732 | 0.369 | 3.924 | 2.079(1.008-4.287) | 0.048 |
| Diabetes (yes) | 0.682 | 0.380 | 3.224 | 1.978(0.939-4.166) | 0.073 |
| SBP | 0.017 | 0.009 | 3.745 | 1.017(1.000-1.034) | 0.053 |
| DBP | 0.022 | 0.010 | 5.423 | 1.023(1.004-1.042) | 0.020 |
| DNT | 0.011 | 0.010 | 1.310 | 1.011(0.992-1.031) | 0.252 |
| INR | -1.084 | 0.852 | 1.620 | 0.338(0.064-1.795) | 0.203 |
| Fg | 0.142 | 0.216 | 0.434 | 1.153(0.755-1.761) | 0.510 |
| TC | 0.304 | 0.230 | 1.760 | 1.356(0.865-2.126) | 0.185 |
| TG | 1.519 | 0.587 | 6.685 | 4.566(1.444-14.441) | 0.010 |
| HDL | -1.017 | 0.517 | 3.869 | 0.362(0.131-0.996) | 0.049 |
| LDL | 0.531 | 0.199 | 7.095 | 1.701(1.151-2.515) | 0.008 |
| HbA1c | 0.105 | 0.106 | 0.978 | 1.111(0.902-1.368) | 0.323 |
| FPG | 0.198 | 0.110 | 3.234 | 1.220(0.982-1.514) | 0.072 |

SBP: Systolic blood pressure; DBP: Diastolic blood pressure; DNT: door to needle time; INR: International normalized ratio; Fg: Fibrinogen; TC: Total cholesterol;TG: Total triglycerides; HDL: High-density lipoprotein cholesterol; LDL: Low-density lipoprotein cholesterol; HbA1c: Haemoglobin A1c; FPG: Fasting plasma glucose.

**Table S2B Model parameter table in optimized model of 30 days after intravenous thrombolysis**

| **Variable** | ***β*** | ***S.D*** | ***Wald χ²*** | ***OR(95%CI)*** | ***P-value*** |
| --- | --- | --- | --- | --- | --- |
| Intercept | -13.456 | 2.534 | 28.205 | - | <0.001 |
| Age | 0.052 | 0.016 | 10.311 | 1.053（1.021-1.088） | 0.001 |
| Smoking (yes) | 0.723 | 0.359 | 4.057 | 2.061（1.020-4.167） | 0.044 |
| Diabetes (yes) | 0.741 | 0.371 | 3.990 | 2.097（1.014-4.338） | 0.046 |
| SBP | 0.018 | 0.008 | 4.856 | 1.018（1.002-1.034） | 0.028 |
| DBP | 0.025 | 0.009 | 7.343 | 1.025（1.007-1.044） | 0.007 |
| TG | 1.741 | 0.563 | 9.546 | 5.701（1.890-17.199） | 0.002 |
| HDL | -1.170 | 0.497 | 5.552 | 0.310（0.117-0.821） | 0.018 |
| LDL | 0.693 | 0.210 | 10.894 | 2.000（1.325-3.020） | 0.001 |
| FPG | 0.270 | 0.101 | 7.222 | 1.311（1.076-1.596） | 0.007 |

SBP: Systolic blood pressure; DBP: Diastolic blood pressure; TG: Total triglycerides; HDL: High-density lipoprotein cholesterol; LDL: Low-density lipoprotein cholesterol; FPG: Fasting plasma glucose.

**Table S3A Model parameter table in multiple logistic regression model of 90 days after intravenous thrombolysis**

| **Variable** | ***β*** | ***S.D*** | ***Wald χ²*** | ***OR(95%CI)*** | ***P-value*** |
| --- | --- | --- | --- | --- | --- |
| Intercept | -17.859 | 3.594 | 24.693 | - | <0.001 |
| Age | 0.050 | 0.019 | 7.048 | 1.051(1.013-1.090) | 0.008 |
| Smoking (yes) | 1.052 | 0.552 | 3.637 | 2.864(0.971-8.444) | 0.057 |
| Drinking (yes) | 0.002 | 0.542 | 0.000 | 1.002(0.347-2.899) | 0.997 |
| Hypertension (yes) | 0.106 | 0.484 | 0.048 | 1.112(0.431-2.871) | 0.826 |
| Diabetes (yes) | 1.103 | 0.456 | 5.856 | 3.012(1.233-7.357) | 0.016 |
| SBP | 0.011 | 0.010 | 1.261 | 1.011(0.992-1.030) | 0.262 |
| DBP | 0.033 | 0.011 | 8.844 | 1.034(1.011-1.057) | 0.003 |
| DNT | 0.005 | 0.011 | 0.205 | 1.005(0.984-1.027) | 0.651 |
| INR | -0.958 | 0.818 | 1.374 | 0.384(0.077-1.904) | 0.241 |
| Fg | 0.387 | 0.248 | 2.434 | 1.473(0.906-2.396) | 0.119 |
| TC | 0.483 | 0.262 | 3.396 | 1.620(0.970-2.707) | 0.065 |
| TG | 2.782 | 0.712 | 15.285 | 16.155(4.005-65.172) | <0.001 |
| HDL | -1.310 | 0.583 | 5.048 | 0.270(0.086-0.846) | 0.025 |
| LDL | 0.617 | 0.247 | 6.233 | 1.853(1.142-3.007) | 0.013 |
| HbA1c | 0.273 | 0.124 | 4.867 | 1.315(1.031-1.676) | 0.027 |
| FPG | 0.234 | 0.120 | 3.822 | 1.263(0.999-1.597) | 0.051 |

SBP: Systolic blood pressure; DBP: Diastolic blood pressure; DNT: door to needle time; INR: International normalized ratio; Fg: Fibrinogen; TC: Total cholesterol;TG: Total triglycerides; HDL: High-density lipoprotein cholesterol; LDL: Low-density lipoprotein cholesterol; HbA1c: Haemoglobin A1c; FPG: Fasting plasma glucose.

**Table S3B Model parameter table in optimized model of 90 days after intravenous thrombolysis**

| **Variable** | ***β*** | ***S.D*** | ***Wald χ²*** | ***OR(95%CI)*** | ***P-value*** |
| --- | --- | --- | --- | --- | --- |
| Intercept | -15.648 | 2.602 | 36.166 |  | <0.001 |
| Age | 0.055 | 0.018 | 9.960 | 1.057(1.021-1.094) | 0.002 |
| Smoking (yes) | 1.086 | 0.382 | 8.087 | 2.962(1.401-6.260) | 0.004 |
| Diabetes (yes) | 1.113 | 0.409 | 7.411 | 3.043(1.366-6.782) | 0.006 |
| DBP | 0.032 | 0.010 | 10.137 | 1.033(1.012-1.053) | 0.001 |
| TG | 2.767 | 0.674 | 16.829 | 15.908(4.241-59.661) | <0.001 |
| HDL | -1.437 | 0.537 | 7.169 | 0.238(0.083-0.680) | 0.007 |
| LDL | 0.994 | 0.272 | 13.316 | 2.702(1.584-4.607) | <0.001 |
| HbA1c | 0.277 | 0.117 | 5.601 | 1.319(1.049-1.658) | 0.018 |
| FPG | 0.294 | 0.109 | 7.286 | 1.342(1.084-1.661) | 0.007 |

DBP: Diastolic blood pressure; TG: Total triglycerides; HDL: High-density lipoprotein cholesterol; LDL: Low-density lipoprotein cholesterol; HbA1c: Haemoglobin A1c; FPG: Fasting plasma glucose.

**Table S4 Multicollinearity detection in multiple logistic regression model of nomogram**

| **Variable** | **TOL** | **VIF** |
| --- | --- | --- |
| Age | 0.953 | 1.050 |
| Smoking | 0.886 | 1.129 |
| Diabetes | 0.853 | 1.172 |
| SBP | 0.905 | 1.105 |
| DBP | 0.941 | 1.063 |
| TC | 0.879 | 1.137 |
| HDL | 0.947 | 1.056 |
| LDL | 0.923 | 1.083 |
| HbA1c | 0.951 | 1.051 |
| FBG | 0.906 | 1.103 |

TOL, Tolerance; VIF, Variance inflation factor

A VIF value more than 10 or a TOL less than 0.1 indicate multicollinearity.


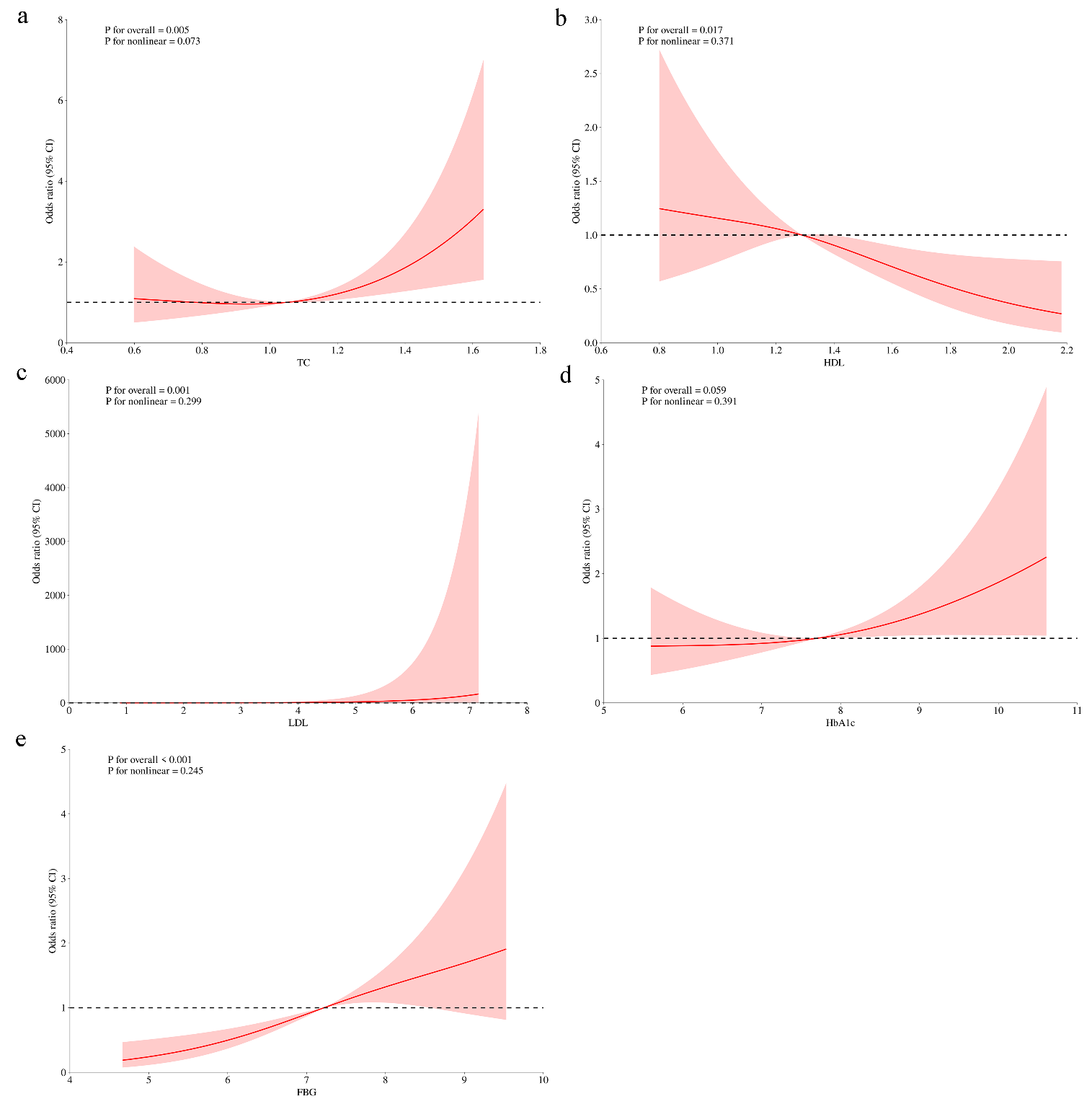


FigureS1 RCS curve of the association between TC(A), HDL(B), LDL(C), HbA1c(D), FBG(E) and prognosis at 30 days.


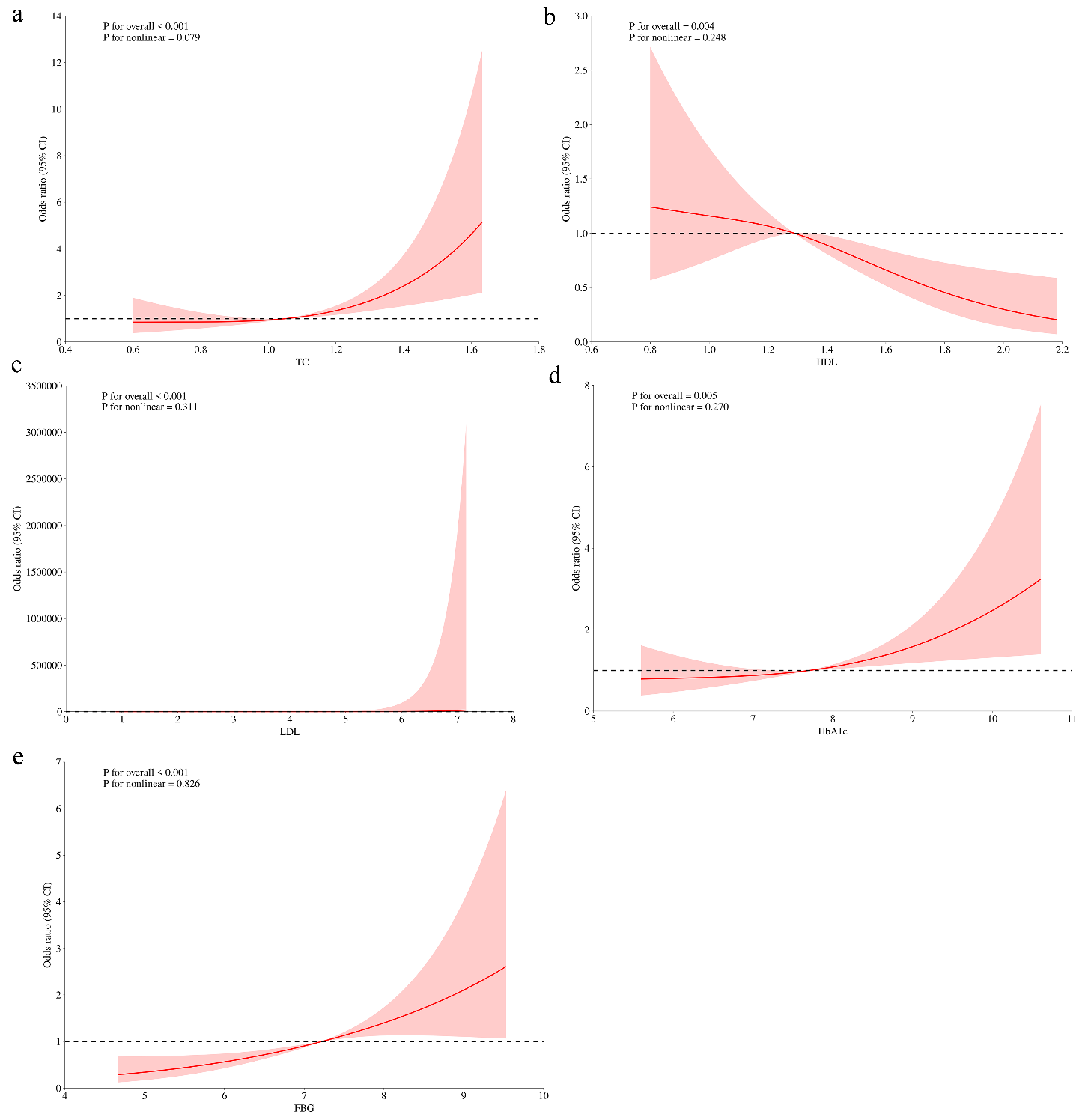


FigureS2 RCS curve of the association between TC(A), HDL(B), LDL(C), HbA1c(D), FBG(E) and prognosis at 90 days.
